# Supplementary material for: The genome sequence of the Purple Treble-bar, Aplocera praeformata (Hübner, 1826) (Lepidoptera: Geometridae)
Source: Wellcome Open Res. 2026 Jan 16;11:31. [Version 1] doi: 10.12688/wellcomeopenres.25721.1 (PMC13291750; doi:10.12688/wellcomeopenres.25721.1)
Supplement: Peer review history

# Reviewer report: Reviewer response for version 1

**DOI:** 10.21956/wellcomeopenres.28319.r146876

**Published:** 2026-06-23

**Authors:** Gulab D Khedkar

**Affiliations:** 1 Dr Babasaheb Ambedkar Marathwada University, Aurangabad, Maharashtra, India

This manuscript presents a high-quality chromosome-level genome assembly of *Aplocera praeformata*, generated using PacBio HiFi and Hi-C sequencing technologies. The study is technically robust, and the assembly meets current reference genome standards with excellent contiguity, completeness, and consensus quality. The methodological workflow is comprehensive, transparent, and highly reproducible, making this genome resource valuable for future studies on Lepidopteran evolution, comparative genomics, and chromosome biology. 

 The manuscript is generally well organized and clearly written. The sequencing strategy, assembly pipeline, quality assessment, and data availability are described in sufficient detail, and the authors provide extensive supporting metrics demonstrating the reliability of the assembly. The inclusion of Merian element analysis further enhances the resource's biological relevance. 

 Optional minor points could further improve the manuscript’s utility:

*The manuscript would also benefit from a short statement outlining future directions, particularly regarding functional genome annotation and comparative genomic analyses.*

**Recommendation:** Accept for indexing in its present form. The manuscript reports a valuable, high-quality genomic resource generated using state-of-the-art sequencing and assembly approaches, and the data will be of considerable interest to the genomics and Lepidoptera research communities.

Are sufficient details of methods and materials provided to allow replication by others?

Yes

Is the rationale for creating the dataset(s) clearly described?

Yes

Are the datasets clearly presented in a useable and accessible format?

Yes

Are the protocols appropriate and is the work technically sound?

Yes

Reviewer Expertise:

Genomics, population genetics, DNA Barcoding

I confirm that I have read this submission and believe that I have an appropriate level of expertise to confirm that it is of an acceptable scientific standard.

# Reviewer report: Reviewer response for version 1

**DOI:** 10.21956/wellcomeopenres.28319.r145119

**Published:** 2026-02-02

**Authors:** Aqeel Alyousuf

**Affiliations:** 1 University of Basrah, Basrah, Iraq

The Methods section is thorough and clearly documented, covering all key stages of the workflow, including sample collection, DNA extraction, library preparation, sequencing, assembly, curation, and quality assessment. The use of established protocols alongside PacBio HiFi and Arima Hi-C technologies demonstrates a high technical standard. The inclusion of detailed information on fragment size, DNA concentration, and purity is particularly helpful for reproducibility.

Are sufficient details of methods and materials provided to allow replication by others?

Yes

Is the rationale for creating the dataset(s) clearly described?

Yes

Are the datasets clearly presented in a useable and accessible format?

Yes

Are the protocols appropriate and is the work technically sound?

Yes

Reviewer Expertise:

Entomology

I confirm that I have read this submission and believe that I have an appropriate level of expertise to confirm that it is of an acceptable scientific standard.
